# Implementation and Rationale for a Unified Clinical and Imaging Protocol for Evaluation and Treatment of Moyamoya Angiopathy: A Single Institutional Experience

**DOI:** 10.3389/fneur.2021.662393

**Published:** 2021-05-17

**Authors:** Anthony S. Larson, Vance T. Lehman, Luis E. Savastano, Giuseppe Lanzino, Norbert G. Campeau, Kirk M. Welker, James P. Klaas

**Affiliations:** ^1^Department of Radiology, Mayo Clinic, Rochester, MN, United States; ^2^Department of Neurosurgery, Mayo Clinic, Rochester, MN, United States; ^3^Department of Neurology, Mayo Clinic, Rochester, MN, United States

**Keywords:** moyamoya, revascularization, vessel wall imaging, cerebrovascular reactivity, BOLD, protocol

## Abstract

Moyamoya disease (MMD) is a complex and incompletely-understood cerebrovascular pathological entity that requires thorough clinical and imaging evaluation. Moyamoya is rare, thereby making the establishment of an effective, thorough and interdisciplinary patient evaluation protocol challenging, even within specialized referral centers. Nevertheless, implementation of such a protocol is crucial in order to provide the best possible evaluation and treatment for MMD patients. Here, we describe our institution's implementation of, rationale for, and experience with a comprehensive multidisciplinary collaboration and evaluation strategy for adult patients with moyamoya. This evaluation course consists of, first of all, a thorough clinical and laboratory evaluation with a vascular neurologist. This is followed by a comprehensive imaging assessment which evaluates angiographic and parenchymal features, in addition to cerebrovascular functionality. Finally, appropriate referrals are made to consulting services as indicated, which includes vascular neurosurgery. These steps are described in detail herein.

## Introduction

Moyamoya disease (MMD) is an idiopathic steno-occlusive vasculopathy affecting the intracranial arteries with formation of fragile collateral neo-vessels (1). MMD is distinct from moyamoya syndrome (MMS), in which patients have a similar radiographic appearance of blood vessel narrowing, but caused by different mechanisms than the genetic mutations that leads to MMD (2). Despite important discoveries relating to the genetic underpinnings of MMD in recent years, a complete picture of the underlying pathophysiology remains to be uncovered (3–6).

Contemporary evaluation of moyamoya vasculopathy involves assessing for the presence of underlying conditions associated with MMS rather than MMD. Next is assessing the risk of ischemic or hemorrhagic intracranial events to determine whether preventative therapeutic measures are indicated. Many different imaging modalities are available for assessment of the severity and extent of MMD, including conventional angiography, various MRI and MRA techniques, CT and CTA techniques, and nuclear medicine studies. These include both anatomic and functional methods, assessing the caliber of the vessels, degree of collateral circulation, brain perfusion, and determining cerebral vascular reserve. The precise imaging techniques employed vary considerably amongst, and sometimes within, institutions. Information gathered from such studies typically guides the decision to address any existing underlying syndromes, pursue medical therapy, or to proceed with surgical revascularization (5).

As the pathomechanisms of MMD and MMS continue to be elucidated, so too does our realization of the complex, and sometimes systemic, nature of these disease processes, including possible involvement of the extracranial internal and external carotid arteries, aorta, pulmonary artery, coronary artery, celiac trunk, and renal artery. As an example, some cases of MMS may require evaluation by a specialist other than a cerebrovascular neurologist or neurosurgeon in order to manage any concomitant extracranial disease. Given our incomplete understanding of the disease as well as the relative rarity, establishment of an evidence-based evaluation and management paradigm remains challenging. It is possible, however, that many existing evaluation paradigms for moyamoya patients are inadequate to address the complexity of the disease. Patients who are ultimately diagnosed with MMD likely require lifelong cerebrovascular care with potential for invasive surgical interventions. It is therefore prudent for specialized centers involved in the care of MMD patients to thoroughly and consistently evaluate each case in order to optimize management strategy.

With a lack of data regarding evaluation and management protocols for moyamoya, descriptions of such protocols from centers with specialized moyamoya care may aid other centers in establishing their own paradigms. Herein, we describe our institution's development of, rationale for and experience with a comprehensive interdisciplinary evaluation strategy for adult MMD patients.

## Multidisciplinary Moyamoya Management and Study Group

**Figure 1:**
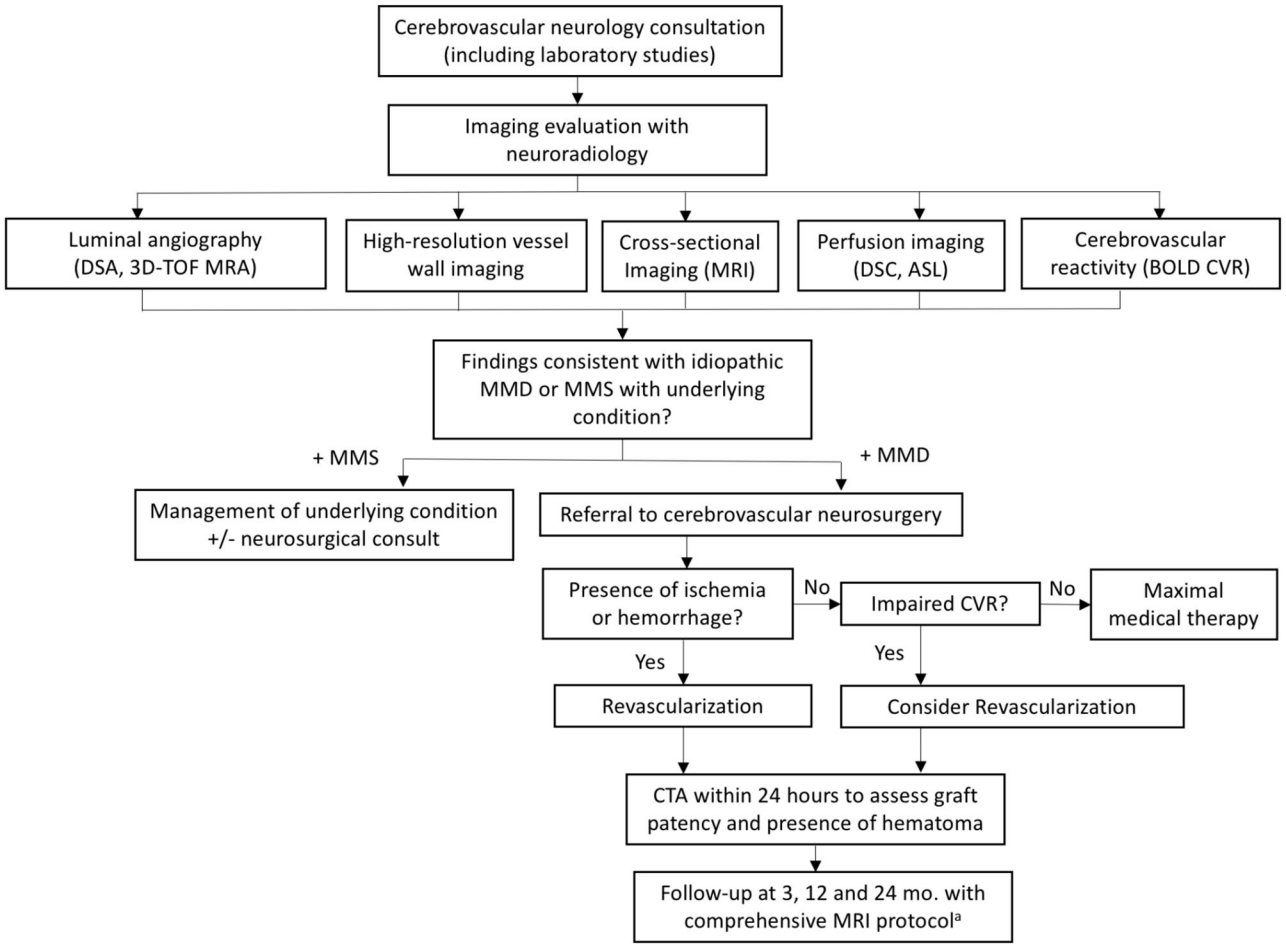
Flowchart summarizing our multidisciplinary evaluation and management process. ^a^Comprehensive protocol includes: 3D TOF MRA, vessel wall imaging, standard brain parenchymal imaging, DSC/ASL perfusion imaging, and BOLD CVR. ASL, arterial spin labeling; BOLD CVR, blood oxygen level dependent cerebrovascular reactivity; DSC, dynamic susceptibility contrast; MMD, moyamoya disease; MMS, moyamoya syndrome; TOF, time of flight.

The first and perhaps most important step taken at our institution was the establishment of a dedicated multidisciplinary moyamoya team including representatives from vascular neurology, vascular neurosurgery, genetics, and neuroradiology. Together, we devised a comprehensive evaluation paradigm to work up or exclude potential causes of moyamoya syndrome, to direct treatment, and to follow patients after conservative or surgical management. This included introduction of a dedicated moyamoya clinic whereby all patients presenting to our institution identified with possible or known moyamoya are evaluated directly by, or occasionally in conjunction with, a dedicated vascular neurologist. This process ensures that patients are evaluated consistently by an experienced clinician and have their work-up streamlined. In conjunction, we standardized and optimized our imaging evaluation. These processes are detailed in the following sections. An outline of our multidisciplinary evaluation and management process is provided in Figure 1.

## Initial Evaluation and Referral to Consulting Services

A key step in evaluating a patient with moyamoya angiopathy is determining whether their overall clinical picture fits more-so with idiopathic MMD, or with MMS. This is determined by an initial evaluation with a vascular neurologist with experience in caring for patients with moyamoya. In cases of MMS, patients will have the presence of an underlying condition that may require evaluation by a specialist in that particular area. MMS is known to be associated with several genetic conditions such as Down syndrome (7), neurofibromatosis type-1 (8) and sickle cell disease, (9) autoimmune conditions including systemic lupus erythematosus (10), Hashimoto's thyroiditis (11) and Grave's disease (12), various prothrombotic conditions (10) as well as connective tissue syndromes such as Marfan syndrome (13). Furthermore, infectious etiologies such as vasculitis, encephalitis, or meningitis may either precede the development of a Moyamoya-like angiographic pattern or be present concurrently as evidenced by lumbar puncture or other laboratory studies (14–17). As such, at the time of initial assessment, all patients undergo standardized serum and CSF testing to screen for these associated conditions (Table 1). As a general rule, all patients will undergo testing with this full panel given that our population consists of a large proportion of North American/Non-Asian patients, and therefore evaluating for mimics or underlying conditions associated with MMS is important. In a small percentage of cases however, individuals may not undergo the complete laboratory workup if a high degree of suspicion is present for idiopathic MMD.

**Table 1:** Blood/serum and CSF tests obtained at initial evaluation to screen for associated conditions.

	**Sample**	
	**Blood/serum**	**Cerebrospinal fluid (CSF)**
**Test**	CBC with differential	Cell count and differential
	Basic metabolic panel	Total protein
	Peripheral smear (morphology evaluation)	Glucose (CSF and serum)
	Thrombophilia profile^*^	Gram stain
	Inflammatory markers (ESR, CRP)	HSV (PCR)
	Connective tissue disease cascade	Parvovirus B19 (PCR)
	ANCA vasculitis panel	VZV (PCR)
	Thyroid function cascade	Oligoclonal banding panel
	Hemoglobin A1c	CSF IgG index panel
	Homocysteine	IgG/Albumin ratio
	Lipid panel	

* *Includes lupus anticoagulant and antiphospholipid antibodies. HSV, herpes simplex virus; PCR, polymerase chain reaction; VZV, varicella zoster virus*.

The concomitance of such conditions with an angiographic and clinical picture of moyamoya requires a balanced care approach, in which the patient is treated by the referring neurologist while at the same time undergoing evaluation by a specialist for the condition that underlies the MMS. For example, patients with underlying diagnoses of neurofibromatosis type-1 and systemic lupus erythematosus would benefit from evaluation by physicians specializing in genetic medicine and rheumatology, respectively. This type of collaboration is crucial, as treating an underlying disease process may improve vascular patency in MMS, therefore obviating the need for revascularization (18). This is particularly true in cases of acquired syndromes such as lupus or antiphospholipid syndrome, whereas chronic, genetic conditions with MMS may eventually require revascularization (7). Our institutional practice therefore relies on early referral to and evaluation by specialists in the specific underlying condition associated with MMS.

## Imaging Evaluation

Imaging studies are crucial for diagnosis, disease staging, and treatment planning. This information, in turn, guides the clinician's decision to pursue medical therapy or to proceed with a revascularization procedure. A neuroradiologist with experience interpreting imaging studies for the specific diagnosis of moyamoya is an essential part of the treatment team. For the purposes of our protocol, imaging assessment of MMD can be classified into five categories: (1) luminal angiography, (2) vessel wall imaging, (3) standard brain parenchymal imaging, (4) perfusion imaging, and (5) assessment of cerebrovascular reactivity. There are several effective techniques to evaluate each of these imaging categories. For our protocol, we consolidated all five imaging categories into a single comprehensive MRI examination. The advantages of this approach includes the relative availability of MRI, the ability to obtain all the required imaging information in a single visit, lack of radiation, and a superior examination of the brain parenchyma. Additionally, among imaging modalities, MRI provides the unique ability to demonstrate important moyamoya features such as the ivy sign and vascular mural abnormalities. This approach also ensures consistent evaluation of all five categories of imaging information by a small group of neuroradiologists familiar with the pulse sequences and the disease. A minor disadvantage of this approach is that the overall scan time is ~90 min, which can be difficult for claustrophobic patients, although we believe that the advantage of consolidating the imaging evaluation into a single appointment offsets this factor. We perform this protocol at our baseline MRI examination and at routine follow-up examinations.

**Figure 2:**
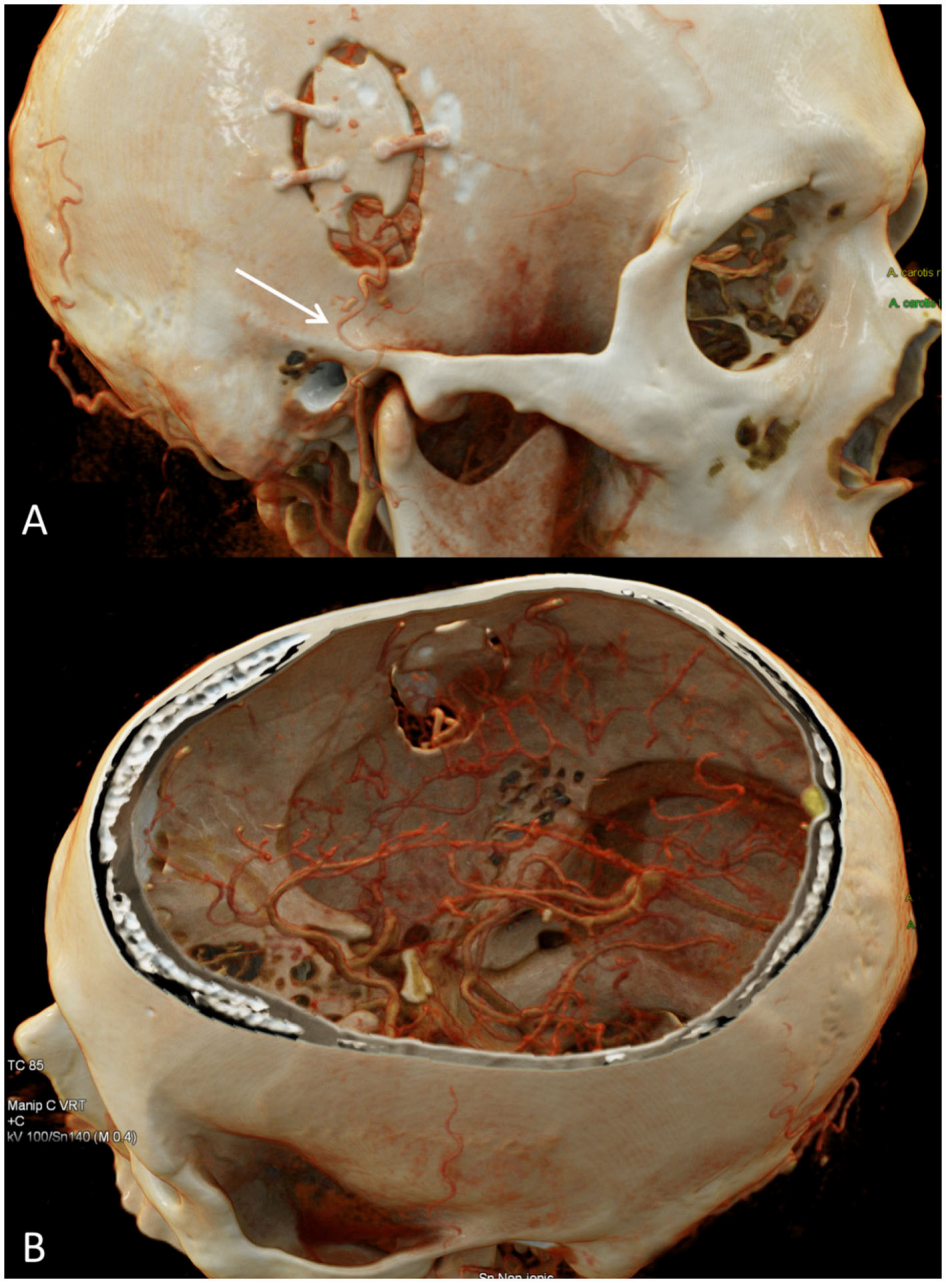
CTA of the head and cinematic rendering provides photorealistic depiction of the STA-MCA bypass postsurgical changes. **(A)** Oblique view of the head shows a right frontal craniotomy with craniotomy flap in satisfactory position. An osseous cut-out within the inferior craniotomy flap permits unobstructed intracranial passage of the STA. There is segmental narrowing of the superficial temporal artery in the vicinity of the zygomatic arch, likely on the basis of vasospasm (arrow). **(B)** Intracranial view with top of calvarium segmented clearly depicts the STA-MCA anastomosis. STA-MCA, superficial temporal artery to middle cerebral artery.

**Figure 3:**
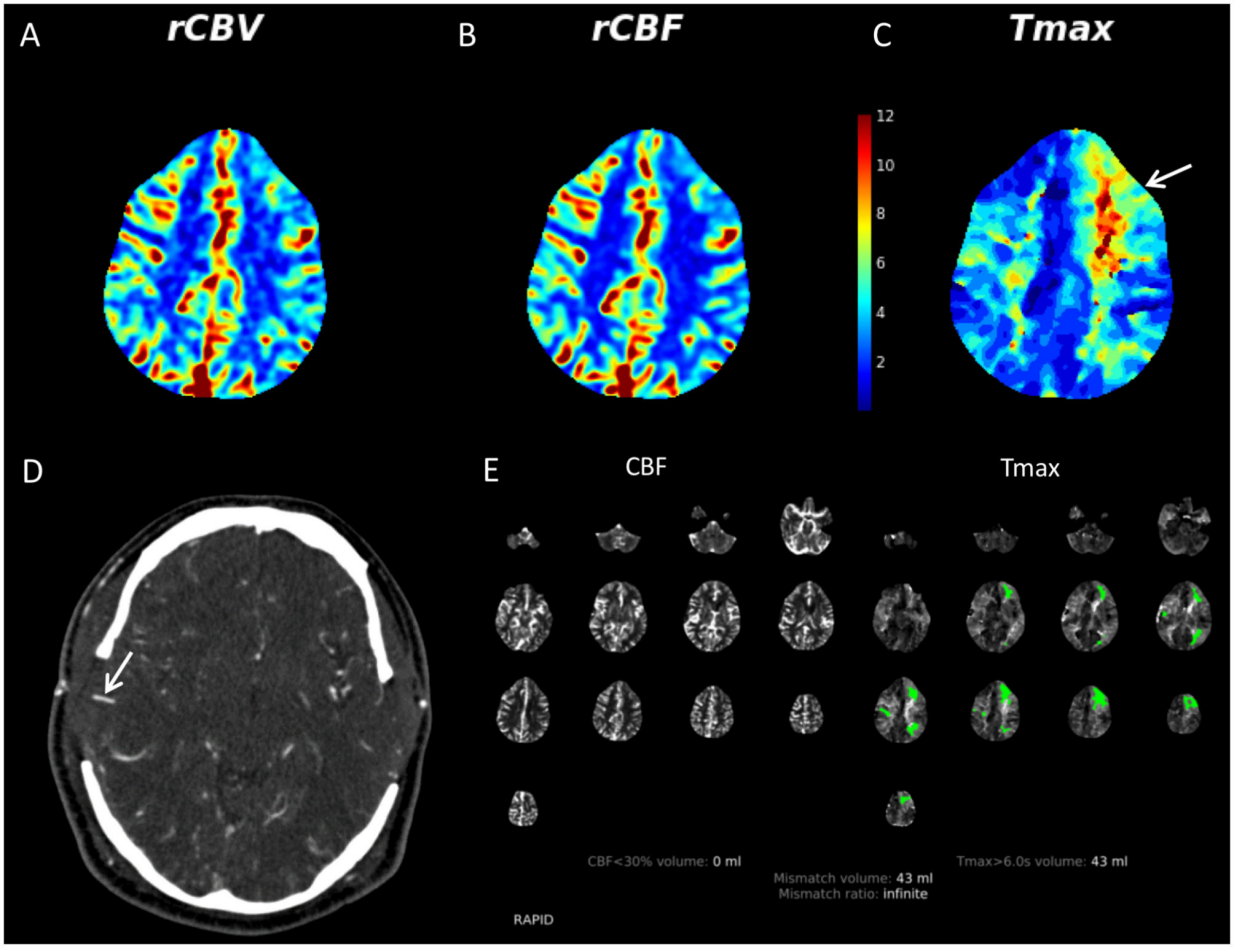
36-year-old male with multiple vascular risk factors presented to our institution with bilateral internal carotid artery terminus narrowing. He had previously undergone a left-sided STA-MCA bypass. He developed aphasia on postoperative day 4 following a right direct STA-MCA bypass (done in conjunction with an indirect right bypass). Given the acuity of the clinical features, the patient was evaluated with CT/CTA/CTP. There was no evidence of acute infarct or hemorrhage on non-contrast CT (not shown). CTP demonstrates mildly increased CBV **(A)** and CBF **(B)** in the right MCA territory compared to the left. There is a region of prolonged Tmax in the left frontal lobe (yellow and red colors, white arrow) **(C)**. Tmax, which represents the timepoint at which the residue of the perfusion deconvolution function reaches its maximum value, has been used in recent years to identify areas of brain parenchyma at risk for infarct (so-called penumbra) in the setting of acute stroke. The CTA demonstrates that both direct bypasses are patent (right shown with white arrow) **(D)**. RAPID software (RapidAI, Menlo Park, CA, USA) indicates that there is no region of CBF <30%, suggesting lack of acute infarct **(E)**. Substantial areas of prolonged Tmax (green regions) suggesting ischemic tissue was present. Lack of acute infarct was confirmed on subsequent MRI. This case demonstrates that it is important to have readily available methods to evaluate acute parenchymal, angiographic, and perfusion changes in patients with MMD as part of a MMD program. CBF, cerebral blood flow; CBV, cerebral blood volume; CTP, CT perfusion; MMD, moyamoya disease; STA-MCA, superficial temporal artery to middle cerebral artery.

Importantly, patients who are referred to our service for evaluation, unless contraindicated, undergo digital subtraction angiography (DSA) in order to interrogate the extent of disease including the degree of arterial stenosis as well as the pattern of basal and leptomeningeal collateralization. Evaluation of these patterns, in addition to determining the diameter and contribution to the intracranial blood supply by various external carotid artery branches enables for planning of potential surgical intervention in cases where it may be indicated. Indeed, DSA is largely considered as the gold standard for evaluation of MMD given its high spatial and temporal resolution (19). Additionally, CT examinations, including CTA and computed tomography perfusion (CTP) may be used in select circumstances such as detailed evaluation of a direct bypass (Figure 2) or emergency evaluations (Figure 3).

## Angiographic Assessment of the Lumen

While patients typically undergo a baseline catheter angiogram, non-invasive approaches are incorporated into our routine follow-up imaging protocol. For MRI examinations 3-D time of flight (3D-TOF) MRA is used. This technique is widely available and allows assessment of the basal arteries, moyamoya collaterals, and direct bypass grafts. CTA offers similar capability but exposes the patient to radiation–an undesirable attribute in a patient population that commonly requires numerous imaging evaluations over a lifetime. Thus, we typically reserve CTA assessment for urgent indications. Physician groups could also consider additional incorporation of a dynamic gadolinium bolus MRA examination such as TRICKS (Time Resolved Imaging of Contrast Kinetics) or TWIST (Time-resolved angiography with Interleaved Stochastic Trajectories) depending on time constraints and local expertise.

## High Resolution Vessel Wall Imaging

High-resolution vessel wall imaging (HR-VWI) consists of a variety of MRI techniques that provide sub-millimeter resolution and suppression of surrounding signal from flowing blood which enables assessment of the arterial wall (20). The most consistent feature of MMD on HR-VWI is negative remodeling of the involved segments (decreased outer wall diameter), although vessel wall enhancement and thickening are also reported (19, 21, 22). The primary utility of HR-VWI lies in diagnosing and distinguishing idiopathic MMD from other disease processes which may appear similar on conventional luminal imaging modalities, such as intracranial atherosclerosis (23). This distinction is beneficial from a management perspective, as patients with idiopathic MMD are more likely to benefit from surgical revascularization, whereas patients with distinct intracranial pathologies (such as atherosclerosis and vasculitis) are typically managed with medical therapy (24). However, neuroradiologist experience is important since the imaging findings such as vessel wall enhancement are variable and could be misinterpreted. Additionally, emerging evidence indicates that vessel wall enhancement may predict disease activity including progression of arterial stenosis and risk for territorial infarct, although the potential role of HR-VWI in this regard requires more data (20, 25).

**Figure 4:**
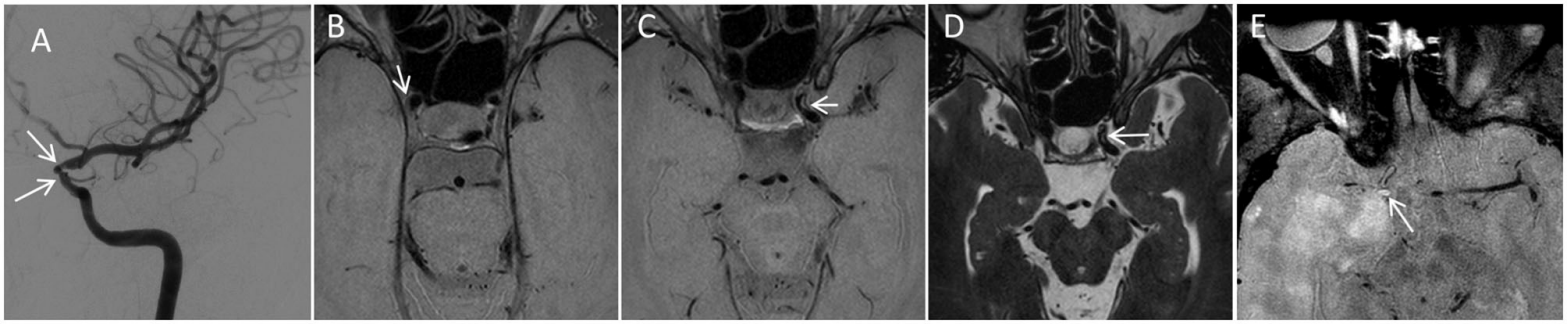
Utility of multicontrast VWI to help establish the etiology of stenosis. A 36-year-old female presented to our institution with areas of bilateral intracranial internal carotid artery (ICA) narrowing, left greater than right. A catheter angiogram from the left ICA injection **(A)** Demonstrates stenosis. The angiographic pattern is atypical for idiopathic moyamoya disease, including stenoses of the clinoid and ophthalmic segments of the left ICA (white arrows) with sparing of the ICA terminus and proximal M1 segments. VWI as part of the comprehensive protocol demonstrated multifocal areas of subtle eccentric vessel wall thickening in both ICAs, Including subtle plaques such one along the lateral wall (white arrow) on axial proton density VWI **(B)**. Eccentric multifocal plaques along the left ICA were more prominent (arrowhead in **C,D**) with rims of non-enhancing intermediate signal on proton density VWI **(C)** and high signal on T2 weighted VWI **(D)**. Such rim of high T2 signal is described as a feature of atherosclerosis. Overall, the anatomic distribution of stenoses, lack of negative mural remodeling, and signal characteristics support atherosclerosis as the etiology. 55-year-old male with a 5-year history of primary CNS vasculitis with prior diffuse segmental involvement of the intracranial arteries **(E)**. He experienced a relapse while on maintenance mycophenolate mofetil, with new stenosis of the right supraclinoid ICA and high-grade circumferential vessel wall enhancement (arrow). While the location and circumferential involvement of the vessel wall are similar to that seen in MMD, the marked degree of unilateral contrast enhancement in conjunction with the clinical scenario are most compatible with vasculitis. ICA, internal carotid artery; VWI, vessel wall imaging.

Given the potential utility for HR-VWI, we include a robust multicontrast vessel wall protocol similar to that described by the University of Washington group (Figure 4) (23, 26). This allows for evaluation of the wall diameter, thickness, contrast enhancement, and T2-weighted features which are thought to help differentiate atherosclerosis, vasculitis, and MMD. Additionally, in our experience, subtle findings such as small areas of atherosclerotic plaque may be more conspicuous on vessel wall imaging than other imaging modalities including 3D-TOF angiography. Incorporation of vessel wall imaging into a standard protocol requires radiologist expertise and is dependent on the availability of requisite MRI hardware and software, although commercially available pulse sequence techniques and experience have become more commonplace in recent years.

When using information from vessel wall imaging examinations to make clinical decisions, it is useful to keep some of the current limitations in mind. First, there is a paucity of pathologic correlation with VWI findings, in particular for MMD. More prospective longitudinal data is needed, including description of findings at initial presentation, over time, and in post-treatment states. Findings may vary amongst the different available VWI techniques, which differ in spatial resolution, methods of blood signal suppression, and other technical aspects. MMD may be a heterogeneous entity with similar radiographic and clinical findings which may depend on the population studied; most publications for MMD are currently from Eastern regions with fewer reports from Western regions including the United States.

## Cross-Sectional Imaging

Standard cross-sectional MRI images allow assessment of numerous important findings including the “ivy sign,” white matter T2 hyperintensity burden, ischemic or hemorrhage infarct, volume loss amongst other findings (19). The precise pulse sequences used will rely on availability and local practice patterns. Our protocol includes standard pulse sequences with an emphasis on high resolution volumetric sequences. For example, volumetric T1 magnetization- prepared rapid acquisition with gradient echo (MPRAGE) allows assessment of T1 characteristics and cerebral volumetric calculations. Volumetric (rather than 2D) T2 fluid-attenuated inversion recovery (FLAIR) images facilitate fusion with blood-oxygen level dependent cerebrovascular reactivity (BOLD CVR) maps and potential for white matter T2 hyperintensity quantification. An echo-planar gradient echo sequence is used instead of a susceptibility-weighted imaging sequence due to time considerations, although some features of MMD may be more conspicuous on the latter (19). Of course, standard diffusion-weighted images (DWI) are obtained in addition to the aforementioned. The primary alternate modality to assess the parenchyma is CT, although MRI is advantageous as it provides more detailed assessment and avoids radiation dose.

## Perfusion Imaging

Well-established techniques useful for characterization of perfusion in MMD include dynamic susceptibility contrast (DSC) perfusion and arterial spin labeling (ASL). Our standardized protocol utilizes DSC perfusion. DSC perfusion is commonly performed in evaluation of glial brain tumors and is a method familiar to MR technologists. The DSC perfusion pulse sequence is performed concomitantly with an intravenously administered gadolinium bolus. In the context of our MMD imaging protocol, patients only require a single bolus of gadolinium for both HR-VWI as well as DSC perfusion which are performed in a single study.

DSC perfusion allows assessment of numerous perfusion parameters: Cerebral blood flow (CBF) enables us to understand if blood delivery to the brain is adequate, mean transit time (MTT), time to peak (TTP), and Tmax aid in understanding the performance of moyamoya induced collateral flow and/or surgically created collateral pathways and their role in maintaining CBF, relative cerebral blood volume (rCBV) helps in understanding the volume of small collateral vessels in any portion of the brain. Together, these parameters create a picture of cerebral hemodynamics when a patient is at their baseline cerebrovascular function.

**Figure 5:**
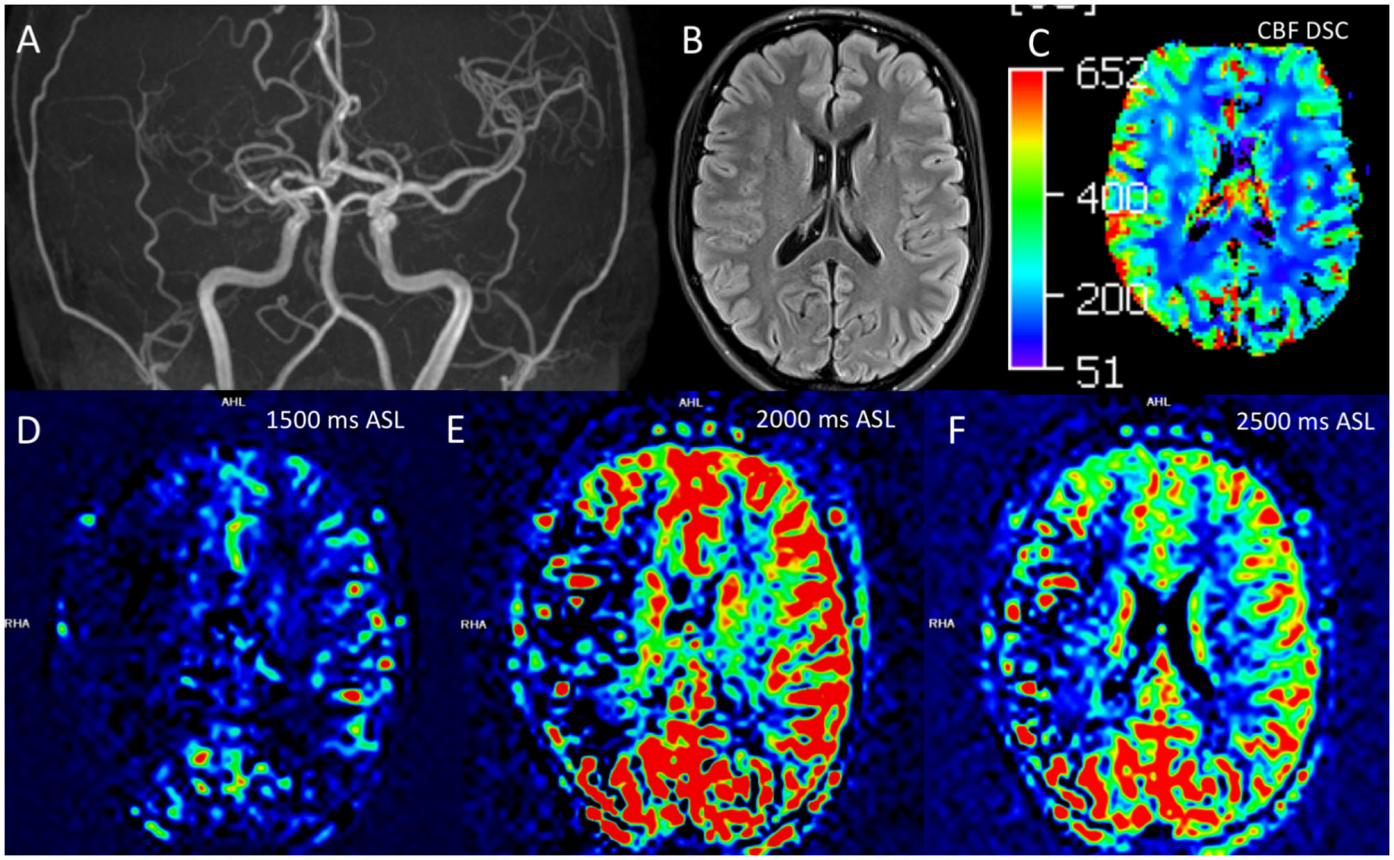
Comparison of ASL and DSC assessments of perfusion in moyamoya disease. Correct interpretation of ASL is heavily dependent on knowledge of the technical parameters and underlying basis for signal generation. A 21 year-old-female had findings of right sided MMD, including stenosis of the right M1, M2, and A1 segments on this coronal 3D-TOF MIP **(A)**. Routine cross-sectional images such as this representative 2D T2 FLAIR image did not demonstrate chronic changes, including lack of infarct, white matter T2 hyperintensity, or Ivy sign **(B)**. DSC perfusion shows preserved or slightly increased CBF in the right cerebral hemisphere **(C)**. ASL perfusion was also obtained with multiple tag delay time points including 1,500 ms **(D)**, 2,000 ms **(E)**, and 2,500 ms **(F)**. The ASL images suggest decreased flow in the right MCA territory; this appearance is more marked at the 2,000 ms timepoint. However, this low ASL signal represented delayed transit through collateral pathways rather than low CBF, demonstrating the ASL must be interpreted in context of the technique and pathophysiology. ASL, arterial spin labeling; CBF, cerebral blood flow; DSC, dynamic susceptibility contrast; FLAIR, fluid-attenuated inversion recovery; MIP, maximal intensity projection; TOF, time of flight.

We will occasionally use ASL perfusion as either a supplement to DSC perfusion or when gadolinium cannot be administered. ASL does not require gadolinium as it relies on extracranial magnetic tagging of inflowing blood as a means of measuring cerebral perfusion. ASL allows assessment of CBF but requires selection of an appropriate post-labeling delay (PLD), which represents the difference in time between “tagging” of inflowing blood and image acquisition. Too short a PLD leads to acquisition before magnetically labeled blood has made its way to the brain, and with an excessively long PLD, there is insufficient labeled blood available to contribute perfusion information. Newer multi-delay ASL techniques are now becoming commercially available and have potential to improve evaluation of processes such as MMD. Decreased perfusion from stenosis itself can lead to exaggerated areas of apparent decreased flow, particularly with short PLD intervals (Figure 5).

There are several other methods to assess perfusion as well which could be considered depending on the clinical scenario, availability, and local expertise. CT perfusion allows assessment of the same parameters, but with the drawback of potentially harmful ionizing radiation exposure. There are also several nuclear medicine techniques including ^99m^Technecium (Tc)-labeled radiopharmaceutical single photon emission computed tomography (SPECT) and ^15^O-water positron emission tomography (PET). ^15^O-water PET has the advantage in that it offers potential for absolute quantification of perfusion rather than just evaluation of relative perfusion in comparison to other brain regions. However, ^15^O-water has a half-life of ~2 min, requiring not just an on-site cyclotron for ^15^O-water production, but also an efficient system for transporting the tracer directly from the cyclotron into the PET exam room (27). Other disadvantages of these modalities include separate appointment times, radiation exposure, and the fact that they are difficult to incorporate into a single unified imaging protocol. If nuclear medicine approaches are employed, it may be useful to have a dedicated representative on a moyamoya treatment team with a solid understanding of the clinical considerations and pathology.

## Analysis of Cerebrovascular Reactivity

**Figure 6:**
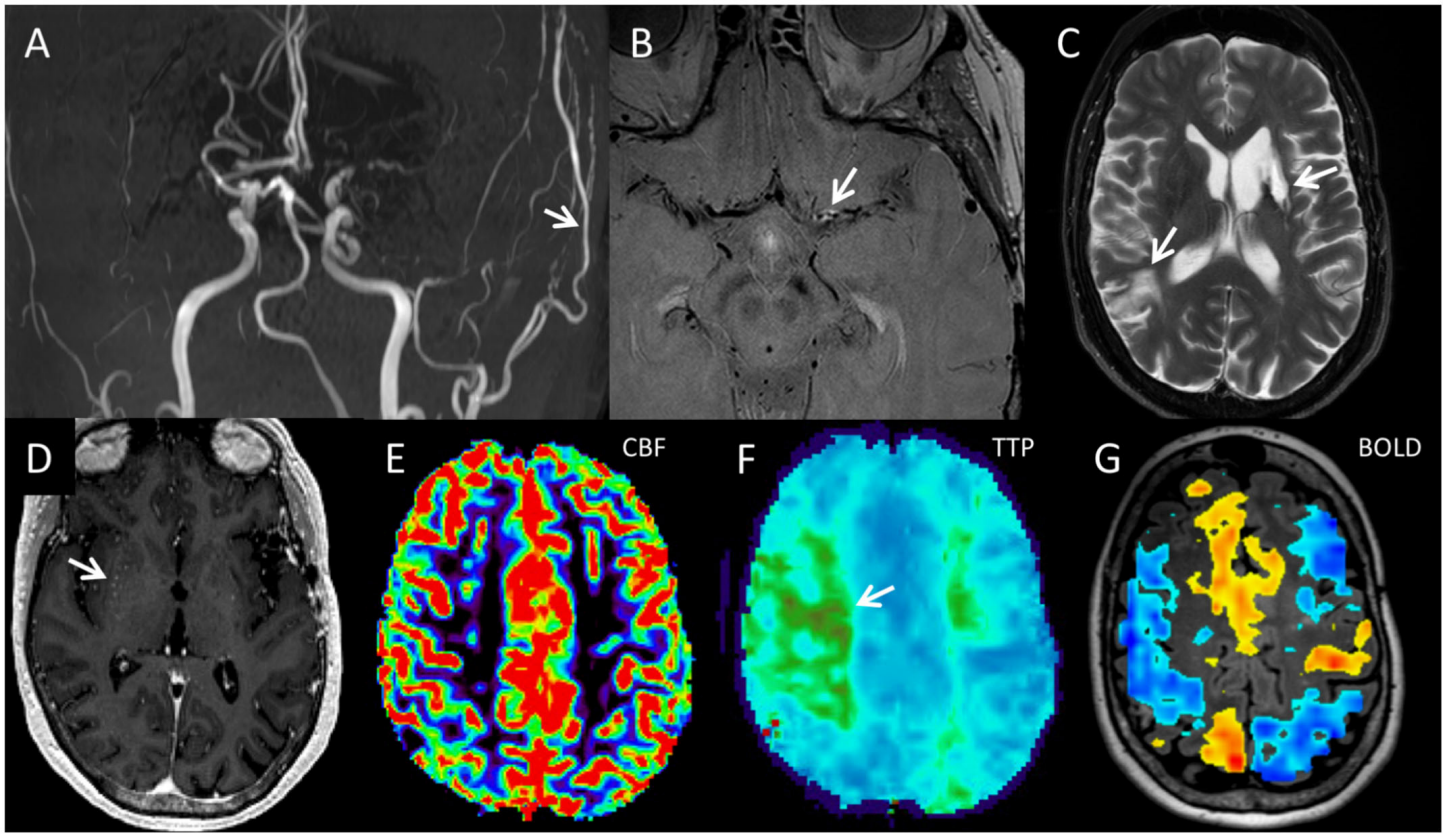
The five components of the comprehensive moyamoya disease imaging protocol include **(A)** luminal angiography, **(B)** vessel wall imaging, **(C,D)** standard cross-sectional imaging, **(E,F)** perfusion imaging, and **(G)** BOLD CVR map, and are illustrated in this 65-year-old female diagnosed with MMD after a comprehensive work-up. Coronal 3D MIP from a 3D-TOF image **(A)** demonstrates narrowing of the bilateral supraclinoid ICAs, M1 segments, and left A1 segment with decreased to absent flow. There is a patent left direct STA-MCA bypass with relatively prominent flow in the left STA (white arrow). Axial proton density vessel wall imaging demonstrates circumferential enhancement of the proximal left M1 segment (white arrow) with negative remodeling **(B)**. Distal to the area of circumferential enhancement, the left M1 segment is narrowed with negative remodeling but no vessel wall enhancement. **(C)** Axial T2-weighted fast spin echo image demonstrates chronic infarcts in the left basal ganglia and right parietal lobe (arrows). **(D)** Axial post-gadoliniumT1 weighted MPRAGE (magnetization prepared gradient echo) image demonstrates dots of enhancement in the right basal ganglia consistent with moyamoya collateral arteries (white arrow). **(E)** Axial DSC CBF images demonstrates preserved blood flow throughout. **(F)** Axial TTP image demonstrates regional delayed transit time within the posterior right frontal lobe and left centrum semiovale region (region of green color, white arrow), consistent with delayed, but preserved flow supplied from collateral moyamoya vessels. A 20 s breath-hold BOLD cerebral vascular reactivity map superimposed on a 3D axial T2 FLAIR image shows large regions of decreased reactivity in both MCA territories including areas that had normal CBF as well as increased reactivity in other regions including the ACA territories. That is, decreased reactivity could either show up on the map as non-activation, which represents decreased reactivity without steal, or blue, which represents decreased reactivity with steal. Areas of steal paradoxically receive less blood during the CO_2_ challenge. Such decreased cerebrovascular reactivity imply lack of hemodynamic reserve in which brain tissue is perfused normally at rest but receive inadequate blood flow when the patient is experiencing a hemodynamic stressor. ACA, anterior cerebral arteries; BOLD CVR, blood oxygen level dependent cerebrovascular reactivity; CBF, cerebral blood flow; CBV, cerebral blood volume; CTP, CT perfusion; DSC, dynamic susceptibility contrast; ICA, internal carotid artery; MMD, moyamoya disease; MPRAGE, magnetization prepared gradient echo; STA-MCA, superficial temporal artery to middle cerebral artery; TTP, time to peak.

Our protocol includes assessment of CVR since this is one factor we consider for recommending surgical revascularization and for following patients after treatment (Figure 6). Analysis of CVR in combination with perfusion imaging is important: Perfusion imaging provides information about a patient's baseline perfusion status but does not provide information regarding cerebral hemodynamics in response to a stressor. Likewise, assessing CVR provides information regarding a patient's cerebrovascular response to a hemodynamic stressor but provides little insight into resting state perfusion. These two modalities performed in combination with one another provide a more complete picture of cerebral hemodynamics at baseline and in context of a stressor.

Again, there are several methods to assess CVR. The most significant change that has taken place over the years at our institution is the utilization of MRI-based BOLD imaging as opposed to SPECT for evaluation of CVR. BOLD CVR imaging is an excellent way to introduce a hypercapnic stimulus and determine if it elicits a vasodilatory response from baseline. It is effectively a way to determine if the underlying vascular tension has been disrupted by pathology. Brain territories that are at risk for infarction due to poor blood flow will have elevated regional carbon dioxide in order to produce local vasodilation and recruit more oxygenated blood. With progressive disease, at some point this compensatory mechanism will no longer be able to keep up and brain function becomes impaired with risk of infarction. BOLD CVR effectively provides a functional assessment of the clinical significance of observed perfusion abnormalities.

The mechanism of BOLD contrast allows imaging of local variability in brain perfusion based on susceptibility differences between paramagnetic deoxygenated hemoglobin and diamagnetic oxygenated hemoglobin. With task-based fMRI examinations (e.g., motor or language tasks), there is overcompensation of arterial inflow increasing weakly diamagnetic oxygenated hemoglobin to metabolically active brain a few seconds after task initiation. This locally increases BOLD T2 signal and provides the contrast for such fMRI examinations. For cerebrovascular reactivity assessment, a 20 s breath hold task can be employed although the onset of BOLD signal increase is significantly delayed in comparison with traditional fMRI tasks. Normal cortical arterioles will dilate in response to increased serum CO_2_ concentration as a result of breath holding, increasing blood flow in brain regions where there is preserved cerebrovascular reserve and thereby causing an increase in BOLD signal. However, arterioles that are already maximally dilated at baseline cannot respond to the CO_2_ challenge and lack the ability to increase local cortical perfusion. Consequently, in regions of brain where blood vessels are maximally dilated, a CO_2_ stimulus will result in reduced or absent BOLD signal. In the worst-case scenario, this may lead to a steal phenomenon in which the CO_2_ stimulus will result in redirection of blood from areas with absent vasoreactivity to areas with preserved vascular reserve. This results in paradoxically negative BOLD signal in the zone(s) of poor vasoreactivity.

The advantages of the breath-hold BOLD cerebral reactivity imaging technique include technical simplicity as well as the ability to easily incorporate this exam as a subcomponent of a larger comprehensive moyamoya MRI exam. However, this technique does require pre-exam coaching, patient compliance, facile MR technologists, and radiologist post-processing time and effort. Also, magnetic susceptibility effects from the skull base impair the ability of this BOLD fMRI technique to evaluate the basal frontal and temporal lobes. Advanced, related BOLD contrast techniques that are theoretically superior to breath-holding have also been used at some facilities. These include gas delivery via mask to enhance the CO_2_ stimulus and computer temporally controlled inhaled gas delivery. However, these methods significantly increase the complexity and time of the MRI examinations and are not as widely available. Another method that is more commonly employed is ^99m^technecium (Tc)-labeled radiopharmaceutical perfusion SPECT with and without administration of acetazolamide (Diamox) to dilate the intracranial arterioles. Disadvantages of this approach include radiation requirement, a lengthier examination, and the need for two separate patient appointments to assess cerebrovascular reactivity.

Transcranial Doppler (TCD) is used to assess vasoreactivity in some Institutions. It is a valid technique but highly operator dependent. The introduction of BOLD MRI in our protocol, has allowed us to assess vasoreactivity during MRI eliminating the need of additional techniques. An additional advantage of the protocol we propose is that the physician interpreting the study has imaging and physiological data available in a single study.

## Surgical Evaluation and Procedures

Although patients treated at high-volume centers are, in general, more likely to have better outcomes as compared to those treated at low-volume centers, this effect is magnified in cases where revascularization procedures are performed (28). All patients who present to our moyamoya clinic with a potential diagnosis of moyamoya receive a referral for neurosurgical evaluation, following which the decision to proceed with a revascularization procedure is made in conjunction with the team members. In recent years, a randomized trial has demonstrated the role for revascularization surgery in patients with hemorrhagic moyamoya (29). While a randomized trial has yet to be performed in regards to the benefit of revascularization for ischemic-type moyamoya, existing literature suggests that revascularization may reduce future ischemic events in such patients (30–33). Based on these data, in addition to recommendations made in published guidelines, patients with hemorrhagic or ischemic events or those with impaired cerebral hemodynamics are considered for revascularization as opposed to conservative management alone (2).

After a patient has been selected for revascularization surgery at our institution, it is necessary to determine which specific revascularization technique is most suited to the particular patient. Revascularization procedures for moyamoya can be categorized into two main subtypes: direct and indirect. Direct cerebral revascularization consists of immediate flow augmentation to the affected hemisphere by supplying an additional source of blood flow via a direct surgical anastomosis between an extracranial donor vessel and intracranial recipient (34).

Indirect revascularization procedures utilize the vascular parasitization capabilities of the hypoxic brain by placing a vascularized graft directly on the pial surface (34). Alternatively, direct and indirect anastomosis may be performed in combination with one another and may have a theoretical advantage to performing either direct or indirect alone: Performing a direct bypass provides immediate hemodynamic improvement, and the indirect bypass improves the midterm result and as a possible fallback strategy in case the direct bypass fails (34). When compared to indirect revascularization alone, combined approaches seem to be superior in terms of improving functional cerebrovascular reserve, angiographic collateralization, as well as reducing the rate of future ischemic events (30, 35).

Although which revascularization technique to utilize should be determined within the context of each unique patient, current literature demonstrates the superiority of direct bypass to indirect bypass in terms of improving angiographic collateralization, functional cerebrovascular hemodynamics, as well as decreasing the rate of post-operative cerebral events (30, 32, 35–38). This pattern seems to be true for pediatric MMD patients as well (31, 39), although, as compared to adults, pediatric patients are more likely to benefit from indirect revascularization procedures alone (36). Given these data, our institutional protocol calls for direct revascularization, either alone or with combined indirect revascularization, in cases of an acceptable donor artery as a first-line option in patients who are surgical candidates. In cases were an undersized or otherwise unacceptable donor artery is not present, indirect revascularization is an acceptable alternative and is considered superior to medical therapy alone.

## Follow-Up Considerations

### Clinical Management

In general, patients are scheduled for follow-up visits 3, 12, and 24 months following a revascularization procedure. The initial follow-up visit consists of a thorough evaluation with the treating neurologist and neurosurgeon. The use of anti-platelets following revascularization suffers from a paucity of data (34). Nevertheless, some data have indicated a potential association of anti-platelets with improved clinical status at mid and long-term follow-up. In these patients, an increased risk of hemorrhagic events was not observed (40). Other data have failed to suggest an association between anti-platelet use and graft patency or ischemic events (40, 41). Despite a lack of robust evidence, our institutional protocol typically utilizes low-dose aspirin in patients who have undergone cerebral revascularization, unless otherwise contraindicated.

### Imaging Assessment

Patients who have undergone revascularization procedures undergo CT and CTA within 24 h following the procedure in order to assess for an extra-axial hematoma (as patients are most often on aspirin) and to assess graft patency. Following discharge after a revascularization procedure, the primary concern is the assessment of bypass graft patency along with development of adequate collateralization. Furthermore, assessing for the presence of interval ischemic events is also important, along with interrogating for expected improvements in cerebrovascular reserve capacity. Our comprehensive MRI protocol allows assessment of these considerations. In addition, duplex ultrasound is first used to examine bypass graft patency and can be used in patients who have undergone either direct or indirect revascularization procedures (42, 43).

Catheter-based DSA is not routinely utilized in follow-up assessments unless the patient is being considered for graft revision, or to plan for contralateral revascularization in cases of bilateral moyamoya.

## Conclusions

Moyamoya patients are likely to have the best outcomes when evaluated and treated at high-volume centers with a specialized care team. Inter-disciplinary collaboration between treating physicians involving the medical, radiological and surgical management of moyamoya patients is crucial in order to offer the best possible care. Here, we describe the development and rational for our institutional adult moyamoya clinical and imaging protocol with the hope that this information may aid other, specialized centers in implementing their own collaborative care protocols. We hope this serves as an example of the value of a multi-disciplinary team and standardized approach. In the future, improved guidelines and increased standardization of approaches amongst institutions would be useful.

## Author Contributions

GL, VL, and JK: study conception. AL: draft writing. AL, VL, LS, GL, NC, KW, and JK: draft editing. All authors contributed to the article and approved the submitted version.

## Conflict of Interest

The authors declare that the research was conducted in the absence of any commercial or financial relationships that could be construed as a potential conflict of interest.

## Abbreviations

ASL: arterial spin labeling
BOLD: blood oxygen level dependent
CBF: cerebral blood flow
CBV: cerebral blood volume
CTP: CT perfusion
CVR: cerebrovascular reactivity
DSC: dynamic susceptibility contrast
DWI: diffusion-weighted imaging
FLAIR: fluid-attenuated inversion recovery
HR-VWI: high resolution vessel wall imaging
MIP: maximal intensity projection
MMD: Moyamoya disease
MMS: Moyamoya syndrome
MPRAGE: magnetization- prepared rapid acquisition with gradient echo
MTT: mean transit time
PET: positron emission tomography
PLD: post label delay
SPECT: single photon emission computed tomography
STA-MCA: superficial temporal artery to middle cerebral artery
SWI: susceptibility-weighted imaging
TOF: time of flight
TTP: time to peak.
